# Hurdles to developing and scaling remote patients’ health management tools and systems: a scoping review

**DOI:** 10.1186/s13643-022-02033-z

**Published:** 2022-08-30

**Authors:** Barimwotubiri Ruyobeza, Sara S. Grobbelaar, Adele Botha

**Affiliations:** 1 Department of Industrial Engineering, Stellenbosch University, Stellenbosch, South Africa; 2 Department of Industrial Engineering, Stellenbosch University, South Africa AND DSI-NRF Centre of Excellence in Scientometrics and Science, Technology and Innovation Policy (SciSTIP), Stellenbosch University, Stellenbosch, South Africa; 3 Department of Industrial Engineering, Stellenbosch University and CSIR Next Generation Enterprises and Institutions, Stellenbosch, South Africa

**Keywords:** Remote patient management, Design framework, Accessibility, Versatility, Integration, Adoption and scalability

## Abstract

**Background:**

Despite all the excitement and hype generated regarding the expected transformative impact of digital technology on the healthcare industry, traditional healthcare systems around the world have largely remained unchanged and resultant improvements in developed countries are slower than anticipated. One area which was expected to significantly improve the quality of and access to primary healthcare services in particular is *remote patient monitoring and management.* Based on a combination of rapid advances in body sensors and information and communication technologies (ICT), it was hoped that *remote patient management tools and systems* (RPMTSs) would significantly reduce the care burden on traditional healthcare systems as well as health-related costs. However, the uptake or adoption of above systems has been extremely slow and their roll out has not yet properly taken off especially in developing countries where they ought to have made the greatest positive impact.

**Aim:**

The aim of the study was to assess whether or not *recent*, relevant literature would support the development of in-community, design, deployment and implementation framework based on three factors thought to be important drivers and levers of RPMTS’s *adoption* and *scalability*.

**Methods:**

A rapid, scoping review conducted on relevant articles obtained from PubMed, MEDLINE, PMC and Cochrane databases and grey literature on Google and published between 2012 and May 2020, by combining a number of relevant search terms and phrases.

**Results:**

Most RPMTSs are targeted at and focused on a single disease, do not extensively involve patients and clinicians in their early planning and design phases, are not designed to best serve a *specific* catchment area and are mainly directed at post-hospital, disease management settings. This may be leading to a situation where patients, potential patients and clinicians simply do not make use of these tools, leading to low *adoption* and *scalability* thereof.

**Conclusion:**

The development of a user-centred, context-dependent, customizable design and deployment framework could potentially increase the *adoption* and *scalability* of RPMTSs, if such framework addressed a combination of diseases, prevalent in a given specific catchment area, especially in developing countries with limited financial resources.

## Introduction and problem statement

The Seventy-first World Health Assembly recognized “the potential of digital technologies to advance the Sustainable Development Goals (SDGs), and in particular to support health systems in all countries in health promotion and disease prevention, and by improving the accessibility, quality and affordability of health services” ([1], p. 1). Among eleven recommendations made to its members were the integration of digital technologies into existing health systems, scale-up, re-use and adaptation of existing digital health systems as well as other relevant tools and identifying priority areas and gaps in research and supporting wide implementation.

One area which was expected to significantly improve the quality of and access to primary healthcare services in particular is “remote patient monitoring and management”*.* Based on a combination of rapid advances in body sensors, artificial intelligence and ICT, it was hoped that remote patient management tools and systems (RPMTSs) would significantly reduce the care burden on traditional healthcare systems as well as health-related costs. However, the uptake or adoption of above systems has remained extremely slow, and as a result, their roll out has not yet taken off especially in developing countries where they could affect the greatest impact [2, 3].

Researchers have identified a number of factors broadly affecting the adoption and scaling of digital health systems [4–6]. These prominently include ease of use, cost-effectiveness, functional efficacy and versatility of addressed diseases as well as system’s integration into existing clinical workflows. To the knowledge of the researchers, there has been so far no comprehensive, integrated framework to help guide RPMTS’ designers, programme developers and technologists in their efforts to plan, design and deploy scalable RPMTSs with the greatest potential for adoption and consistent use [7, 8]. This review explores the potential of such an integrated framework to help RPMTS designers, developers and implementers improve the adoption and scaling of RPMTSs, especially in developing countries.

On the one hand, RPMTSs’ evaluation studies remain thin on the ground. There does not exists enough consistent empirical, compelling evidence to prove that, or evaluate if, the use of above systems and tools necessarily leads to reduced healthcare costs, better quality of care or health outcomes and/or broader, more equitable access to healthcare services [9–13]. On the other hand, a research gap remains in terms of the contextual issues relating to the conception, planning, design and deployment of such systems. Context-specific infrastructural and socio-economic factors affecting the adoption and scalability of above RPMTSs do not seem to receive adequate attention during the conception, planning, design and development phases of most RPMTSs. For example, even though Pinnock and McKinstry ([13], p. 190) found that “successful implementation of telehealth-care programmes in rural and remote settings is contingent upon technical, organisational, social and legal considerations at the individual, community and system levels”, it does not seem that these factors are given priority during the conception, planning and design phases of most RPMTSs. However, successful interventions are more likely to be those, the planning and design of which included the consideration of above factors. For example, Walker et al. ([14], p. 84) concluded that patients’ fear of being lost in data may explain reported limited adoption and long-term adherence to remote monitoring and suggested that “remote monitoring devices may benefit from a user-centred design approach that incorporates the patient preferences, requirements and needs”. Other authors such as [4, 5, 8, 15, 16] highlighted similar challenges.

In addition, it is generally accepted that when a diagnosis is done accurately and early, a patient has the best opportunity for a positive outcome and the benefits of medical prognosis are well documented [17]. Yet, the above diagnosis is only triggered by a patient who experiences a health problem, considers his or her symptoms without the necessary medical expertise and decides to engage with a healthcare system, which may be too late. Furthermore, healthcare facilities currently use different, distinct methods and technologies to detect and diagnose health conditions and diseases. These technologies or methods tend to be generally fragmented, focused on a single health issue or condition, available only at healthcare facilities and are generally reactive rather than pre-emptive in nature. Their equivalent eHealth solutions also seem to be fragmented and often targeted at a single disease or condition [11, 15, 18], mainly focused at specific sections of the population (ex. elderly or young patients) and, as already stated, are often criticized for the persistent lack of adoption and scope for scalability [2, 3].

In response to the above gap, Wickramasinghe and Bodendorf ([16], p. 24) recently posited that in order to realize technology’s full potential in this regard, “it is imperative to understand the healthcare-technology paradigm, develop sustainability models for the effective use of technology in a ‘specific’ context, then successfully design and implement ‘patient-centric’ technology solutions which are sufficiently precise, easy to use and available or accessible to the general public”. Moreover, Straub [19], who reviewed the most prevalent technology adoption theories, made the following three key observations:

- Technology adoption is a complex, inherently social, developmental process
- Individuals construct unique (but malleable) perceptions of technology that influence the adoption process
- Successfully facilitating a technology adoption needs to address cognitive, emotional and contextual concerns

In this paper, we argue that the above conclusions may suggest that in addition to research efforts focusing on assessing barriers and challenges related to functional efficacy and cost-effectiveness of RPMTSs, more research efforts need to be directed towards understanding the specific contexts within which these systems and tools are to be deployed prior to and during the early phases of their design and development, especially in low-income countries, rural settings and other areas with limited financial resources [8, 14]. We assess the potential for a new, process framework to assist practitioners in this area to integrate all these aspects.

Focused research on a particular, specific healthcare context may include aspects related to existing local ICT infrastructure, clinicians and users’ perceptions and attitudes toward proposed RPMTS interventions, potential users’ socio-economic circumstances, RPMTSs’ impact on clinicians current work practices, preferred components, features and uses of RPMTSs and their technical and economic feasibility [20, 21]. Pragmatic studies of this nature could help designers determine the unique, local bundle of health benefits and related cost savings that RPMTSs are likely to deliver to a particular group of users and clinicians in a given demarcated area and context, before or at least during the design and development phases of new RPMTSs [8, 18]. Therefore, the aim of this review is to assess whether the development of a process framework to guide localized, in-community, integrated, RPMTSs’ planning, design and development processes may ultimately help alleviate the current poor adoption and limited scaling of RPMTSs. In the following section, we introduce the methods used for the scoping review (see the ‘Methodology’ section) after which, the findings and results of the review are presented in the ‘Findings and results’ section. We then discuss the implications of our findings in the ‘Analysis and discussion’ section and reflect on the limitations of the study in the ‘Limitation of the review’ section. Finally, we draw our conclusions and recommendations in the ‘Conclusions and future work’ section.

## Methodology

### Focus and study placement

In this review, the researchers followed the Preferred Reporting Items for Systematic reviews and Meta-Analyses extension for scoping reviews (PRISMA-ScR) and adapted it to encapsulate existing and well-known, identified barriers and facilitators to adoption and scaling of RPMTS interventions with the end goal of ultimately assessing the potential of a framework being proposed to alleviate the problem. We report on 18 out of the 22 PRISMA-ScR’s reporting items. However, as suggested by Leonard, de Kock and Bam, barriers and facilitators to the adoption and scaling of RPMTS interventions do not all have an equal impact on the adoption and scaling of RPMTSs as some factors have a much bigger impact than others in this regard [22]. Thus, prior to the scoping review, the researchers reviewed and analysed factors identified in [4, 5, 22] and consolidated them into six major categories to gain an understanding of how they relate to each other as well as the extent to which they may impact RPMTS’ adoption and scaling efforts (see Appendix 1). This examination then served as a foundation for analysing how the proposed framework may help developers enhance facilitators while at the same time overcoming contextual barriers to adoption and scaling of RPMTSs as and when they may arise.

**Fig. 1:**
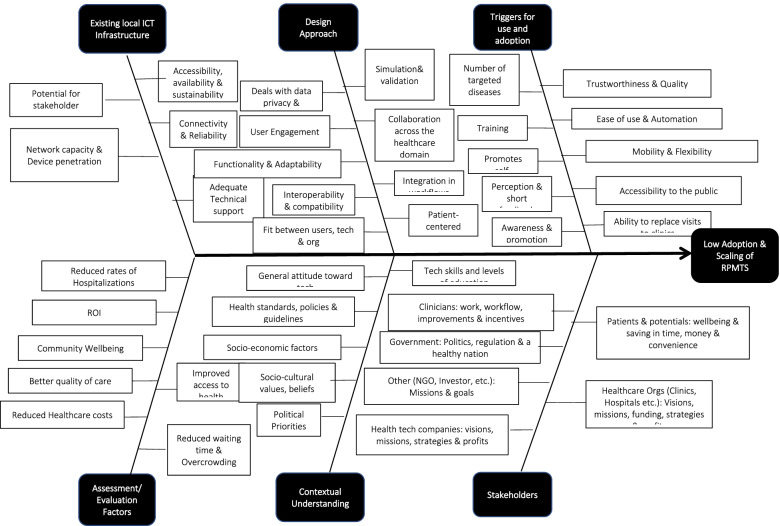
Cause-effect diagram for RPMTSs’ low adoption and limited scope for scaling

The main categories identified included stakeholders’ interests, contextual understanding, existing local ICT infrastructure, design approach and triggers for adoption and use as well as post-deployment assessment factors. The resulting consolidation of above factors is displayed in the cause-effect diagram in Fig. 1.

Given that existing, local ICT infrastructure, stakeholders’ interests and RPMTSs’ post-deployment evaluation factors are all arguably an integral part of ‘contextual understanding’ which ought to be examined before the planning, design and development of new RPMTSs, this review focused on ‘triggers for adoption and use as well as ‘the design approach’ in addition to ‘contextual understanding’. Thus, to reiterate, the three key levers considered to be the most influential in the process of adoption and scaling of RPMTSs in this study are:

- Careful contextual research, prior to the planning, design and development of RPMTS interventions (particularly deep contextual understanding of a catchment area and its existing, local ICT infrastructure)
- Targeting a combination of local diseases, rather than a single disease to increase RPMTS interventions’ reach and accessibility to the general public
- Adopting a user-centred or patient-centric design to facilitate automatic or semi-automatic integration into traditional clinical workflows

Therefore, in order to obtain a general understanding of how a conceptual framework focusing on a combination of local diseases prevalent in a given catchment area, with a patient-centric approach, might help designers and developers improve the adoption and scalability of RPMTSs and consequently increase the quality of and access to primary healthcare services, recent literature on remote patients’ health management tools and systems was accessed and reviewed with a view to assessing their current development and use with regard to the six key variables listed in Table 1.

**Table 1:** Dimension and variables considered

Dimension/variable	Description	Practical relevance to the review
**Intervention’s positioning** within the healthcare domain or industry	Space where the intervention is located (emergency, prevention, primary care, hospital and post-hospital)	Some areas of the healthcare domain may lend themselves to RPMTSs than others (less regulated, more easily conceivable, acceptable or convenient)
Levels of **integration** within existing clinical workflows	The extent to which traditional healthcare facilities are involved or linked with end users (patients and potential patients)	To reduce the care burden on traditional healthcare systems, the intervention has to facilitate service delivery within the community (at health centres, clinics, hospitals or home-based care)
Function **versatility**	Diversity of measured vital signs, symptoms and number of diseases targeted	The greater the number of diseases targeted and the greater the variety of functions (measured vital signs, assessed symptoms) performed by an RPMTS intervention, the better the chances for its adoption and scaling
**Accessibility** to the general public	Availability, affordability and ease of use	The more accessible an RPMTS intervention is, the more adoptable and scalable it is likely to be
Main intervention’s **purpose** set by the owner organization	Prognosis, diagnosis, wellness, monitoring or emergency alerts	The better an RPMTS intervention meets the needs of its owners (healthcare organization), the greater the chances of it being promoted and supported by management and healthcare workers
Main **design approach**	A user-centred approach versus technically and/or otherwise driven	The more the users are involved in the design of an RPMTS intervention, the greater its chances of meeting their needs and, hence, easily adoptable by them

### Eligibility criteria and information sources

The researchers defined article search strategy and parameters. A choice was made to conduct one search but two reviews: one review of systematic reviews and another review of primary articles to confirm the validity of results. First, the inclusion and exclusion criteria were defined for the review of systematic review articles. In addition to being systematic reviews, articles were included if they:

- Involved the use of ICT between healthcare professionals and patients or their representatives
- Had the objective of assessing the adoption or scalability of RPMTSs, increasing access to healthcare services, improving the quality of care and/or reducing healthcare costs
- Related to interventions which integrated into or with traditional healthcare systems (community health centres, clinics and hospitals)

Non-recent systematic review articles were excluded, if they were published before 2012 and did not involve any type of remote patient management intervention or such an intervention was used exclusively within the boundaries of healthcare facilities (through WiFi, LAN and telephone) without the involvement of offsite, end-users (patients, their representatives and potential patients) via the broader ICT infrastructure (WAN, MAN, satellite, GSM and IoT).

Second, the researchers defined additional, but slightly different inclusion and exclusion criteria for primary articles to supplement systematic review articles. Included articles were those which:

- Discussed some use of a mobile/web application by end-users (patients, their representatives and potential patients)
- Their main objective included prognosis, diagnosis or monitoring of diseases and/or prescheduling of visits to healthcare facilities (not just alerts)
- Integrated into or with traditional healthcare systems (communication with healthcare workers to improve service delivery within a healthcare facility)

Primary articles were excluded if they were published before 2014 and did not involve the actual planning, design, development, deployment, implementation or at least the evaluation or some proposal of a remote patient management system or such a system was used exclusively within the boundaries of healthcare facilities (through WiFi, LAN and telephone) without the involvement of end-users (patients, their representatives and potential patients) via the broader ICT infrastructure (WAN, MAN, satellite, GSM and IoT).

To obtain a general overview of the six key variables or angles of the targeted gap by surveying the literary landscape in the field of RPMTSs, one of the researchers looked for relevant articles in PubMed, MEDLINE, PMC, Cochrane databases and grey literature on Google by combining a number of relevant terms and phrases as shown in Table 2.

**Table 2:** Search strategy (with PICO)

		Search terms and phrases
Problem/population	**P**	Remote primary care, potential patients, clinic’s catchment area, remote consultation, telenursing, telemedicine, online systems, primary healthcare, integrated delivery of healthcare and integrated primary care systems
Intervention	**I**	Remote patient monitoring, remote sensing technology, patient health, monitoring systems, integrated system health management, integrated advanced information management systems, development of condition-based management, self-diagnosing AI technology, digital health technologies and patient monitoring system
Comparator (current adoption and scaling of RPMTSs)	**C**	Health information systems, point-of-care systems, clinical decision support systems, health systems plans, systems integration, systems analysis, patient identification systems, data systems and learning health system
Outcome of interest	**O**	Desired (*no search terms*)

### Search, screening and selection of articles

**Fig. 2:**
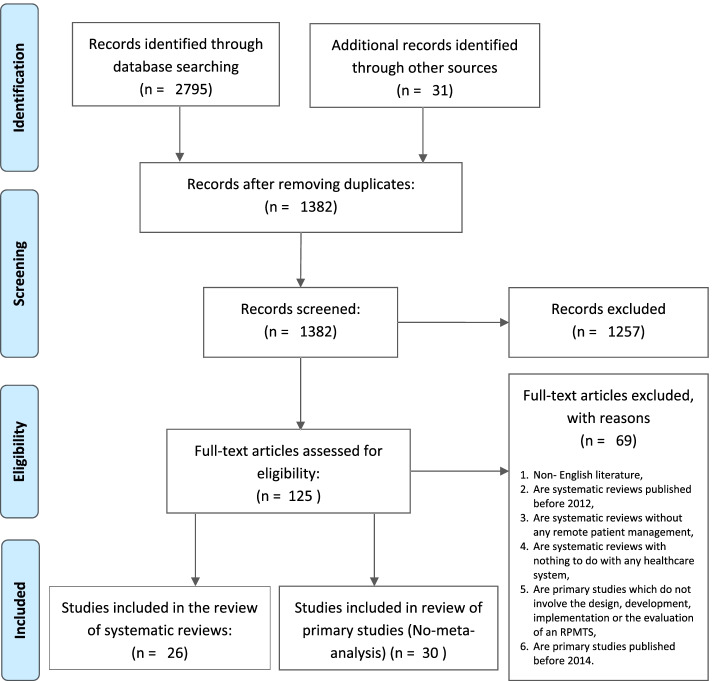
Article search and screening process

As can be seen in Fig. 2, a total of 2826 articles were originally found from the above mentioned databases. Based on titles alone, 1389 articles were considered irrelevant to the subject of interest and 1382 articles remained after removing duplicates. The researcher doing the selection further excluded 1257 articles based on abstract readings and in the end only 125 articles were subjected to full-text review and inclusion and exclusion criteria applied as previously indicated.

In the end, 26 systematic reviews and 30 primary articles were deemed relevant for the scoping review at hand. Sixty-nine articles either did not meet the inclusion criteria or were excluded because they met the exclusion criteria as had been stipulated. The researchers finally focused their attention on the remaining 56 articles, starting with the 26 systematic reviews.

### Data extraction

Each reviewed article was read in full and assessed based on the six variables of interest (position, integration, versatility, accessibility, main purpose and design approach) and the research question being addressed.

As depicted in Table 1, where information relevant to a variable of interest was identified, it was extracted and tabulated for later content analysis to derive dominant patterns and trends which were thought to potentially be relevant in ultimately addressing the topic of interest (see Appendix 2).

## Findings and results

### RPMTS positioning in the healthcare landscape

**Fig. 3:**
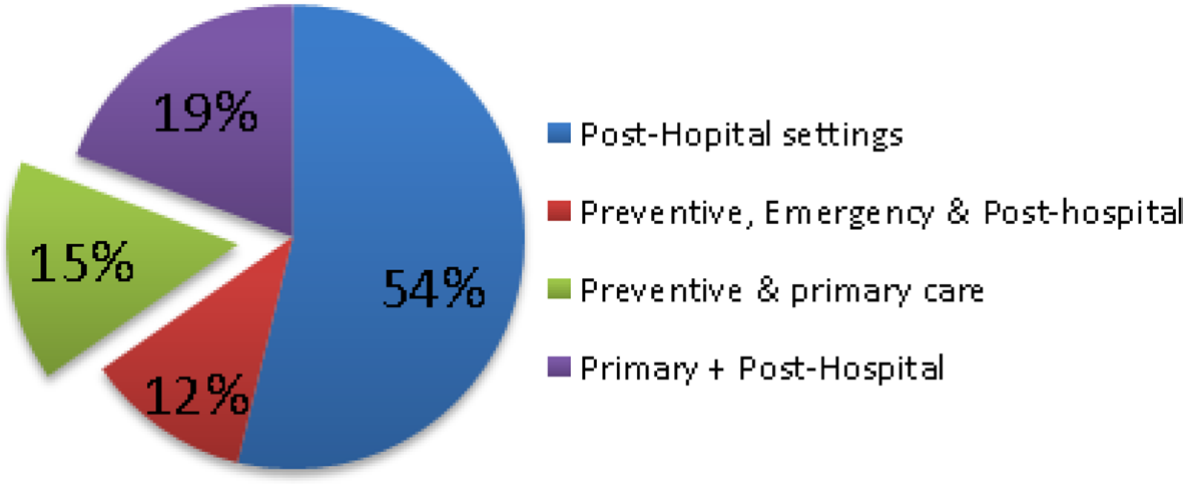
SR-RPMTS deployment in the healthcare domain

Even though many reviews could not neatly fit in one healthcare setting, 14 out of the 26 systematic reviews (SR) included remote monitoring systems positioned in post-hospital care settings. As can be seen in Fig. 3, most of the above systems had been deployed to monitor chronic conditions, previously diagnosed within hospitals and had been deployed in the context of continuity of care including detecting signs of deterioration or improvement in chronic disease, treatment or rehabilitation, patient’s advice, support, education or training, medication adherence and cost reduction in hospitalization.

Ten articles [4, 13, 22–29] dealt with interventions that could be clearly classified as falling into the preventive, pre-clinical or hospital, emergency and/or primary care settings. The majority of the above articles, except [22, 25, 28], combined the above setting with other settings such as the hospital or post-hospital monitoring. And as can be seen in Fig. 3, four articles [4, 13, 23, 24] were both in the preventive and primary care settings without the involvement of any hospital or post-hospital monitoring.

**Fig. 4:**
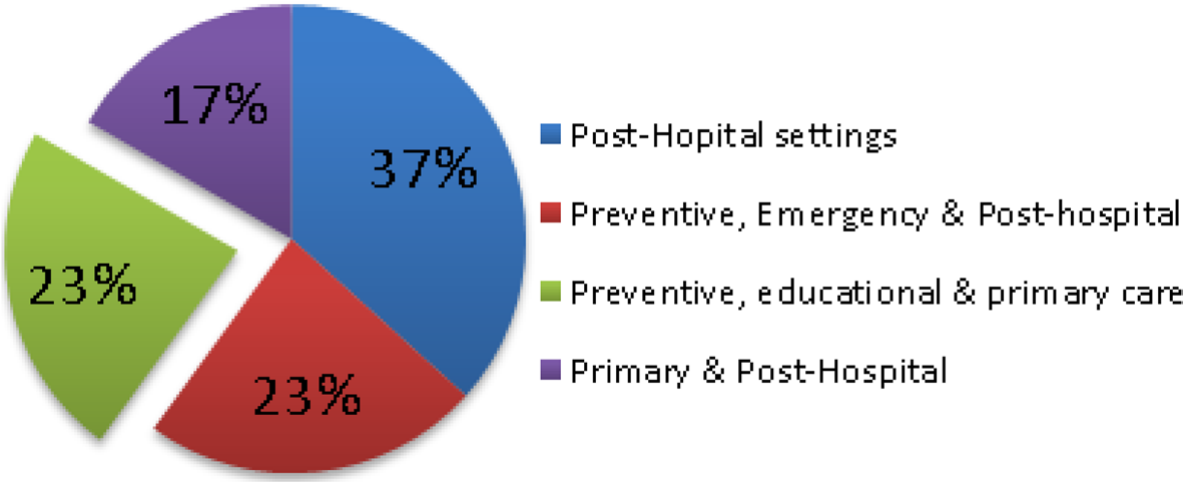
PA-RPMTS deployment in the healthcare domain

For the 30 primary articles (PA) considered, 12 articles [3, 30–40] were classified as falling into the preventive, pre-clinical or pre-hospital, emergency and/or primary care settings of the healthcare landscape. Eight of the above mentioned 12 articles [30, 32–37, 40] combined the above setting with other settings such as the emergency, hospital or post-hospital monitoring. Five articles [3, 31, 34, 35, 37] discussed interventions in which preventative measures were implemented in primary care settings and two additional interventions [38, 39] were set in the educational, preventive, pre-primary settings. Therefore, as demonstrated in Fig. 4, a total of 7 articles were both in the preventive and primary care settings.

### Levels of integration within traditional healthcare systems or facilities

**Fig. 5:**
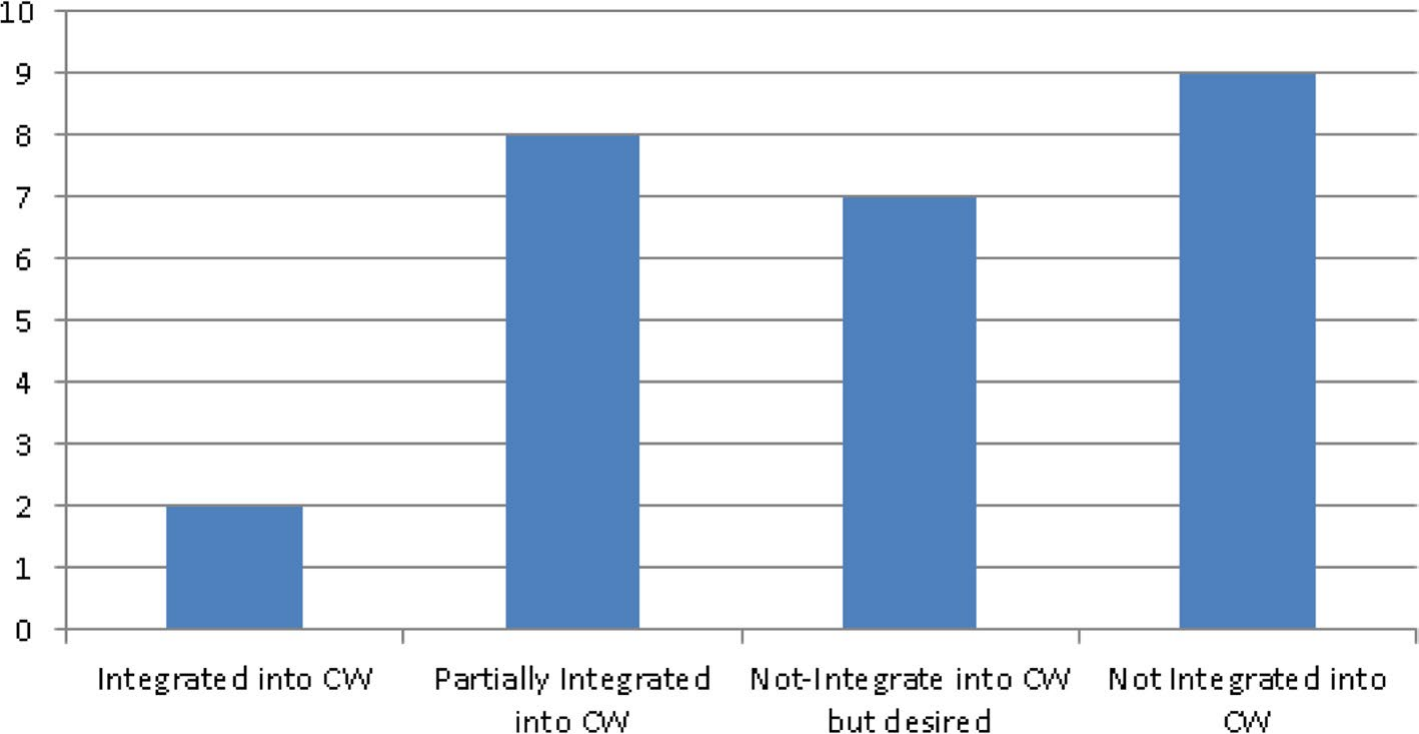
SR spread of integration into CWF

Integration here refers to the degree to which an intervention facilitates existing clinical work or improves existing clinical workflows [5, 28]. A significant number of systematic reviews (nine) did not contain information about the extent to which discussed interventions integrated or intended to integrate with/into traditional healthcare systems. Of the 26 reviews, 9 [4, 10, 13, 24, 25, 41–44] provided information to suggest or demonstrate that above integration was either achieved or at least attempted. Furthermore, in the majority of above 9 cases, ‘integration’ simply meant communication with one or more healthcare professionals via phone or video conferencing and not necessarily integration into clinical workflows. This spread can be viewed in Fig. 5.

In the remaining 8 reviews [2, 5, 12, 23, 28, 29, 45, 46], discussed interventions were partially integrated into traditional healthcare systems and highlighted challenges which hampered complete integration into clinical workflows. For example, one review [28] identified two key barriers to integration: the ‘diversity of available technologies’ and ‘lack of comprehensive guiding framework for standardizing data collection and integration’. Another review [2] pointed to the use of non-scalable and silo solutions which suffer from the absence of interoperability and clinical acceptance to facilitate user engagement and self-management of chronic diseases. Other reviews highlighted additional concerns affecting integration with traditional healthcare systems. One of the above reviews [5] indicated that healthcare practitioners view some aspects of mHealth as negatively impacting their credibility and autonomy and thus hampering their acceptance of such tools and systems, while another [29] highlighted the lack of integration of community-based health information systems (IS) in formal national health management IS (without complete integration, there are duplicative efforts in data collection, analysis and reporting) and the lack of technical capacity of community workers. Finally, review [47] observed that despite the huge research effort on remote care technology, there has not been a sufficient number of successful interventions which have gone past the research environment, broadly taken up and routinely used in clinical settings.

**Fig. 6:**
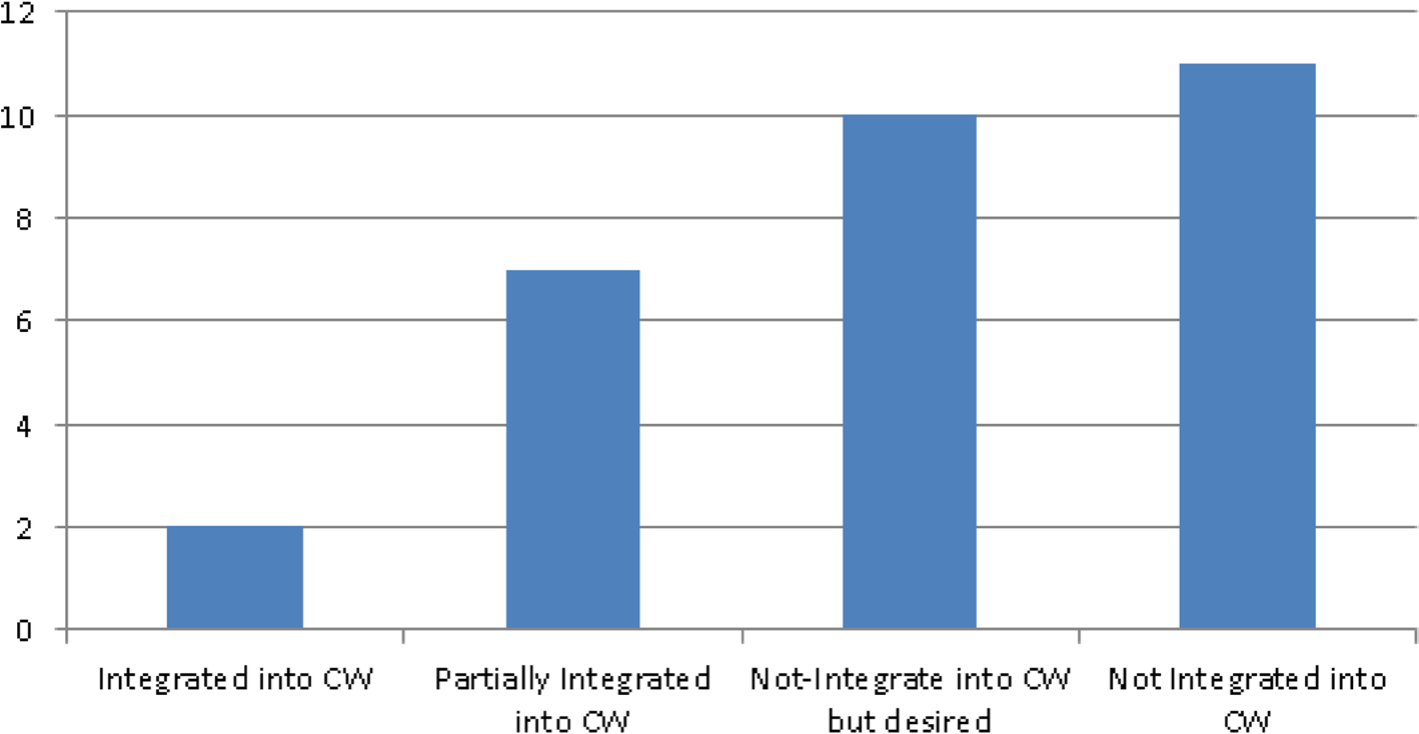
PA spread of integration into CWF

With regard to primary articles, the picture was similar. Eleven articles did not provide sufficient details to determine the extent to which considered interventions integrated or intended to integrate into traditional healthcare systems. As demonstrated in Fig. 6, in 10 of the 30 articles [20, 31, 35, 36, 47–52], there was sufficient information to establish that integration with traditional healthcare facilities had at least been considered. Again, in most cases above, integration was limited to audio or video communication with healthcare providers or some alert mechanisms. Nine of the 30 articles [18, 32, 38, 40, 53–57] provided evidence of limited integration into traditional healthcare systems and three in particular [18, 53, 55] highlighted the significant potential that could be realized if discussed interventions were integrated into existing EMR/EHR.

### Functional versatility (number and nature of targeted diseases)

**Fig. 7:**
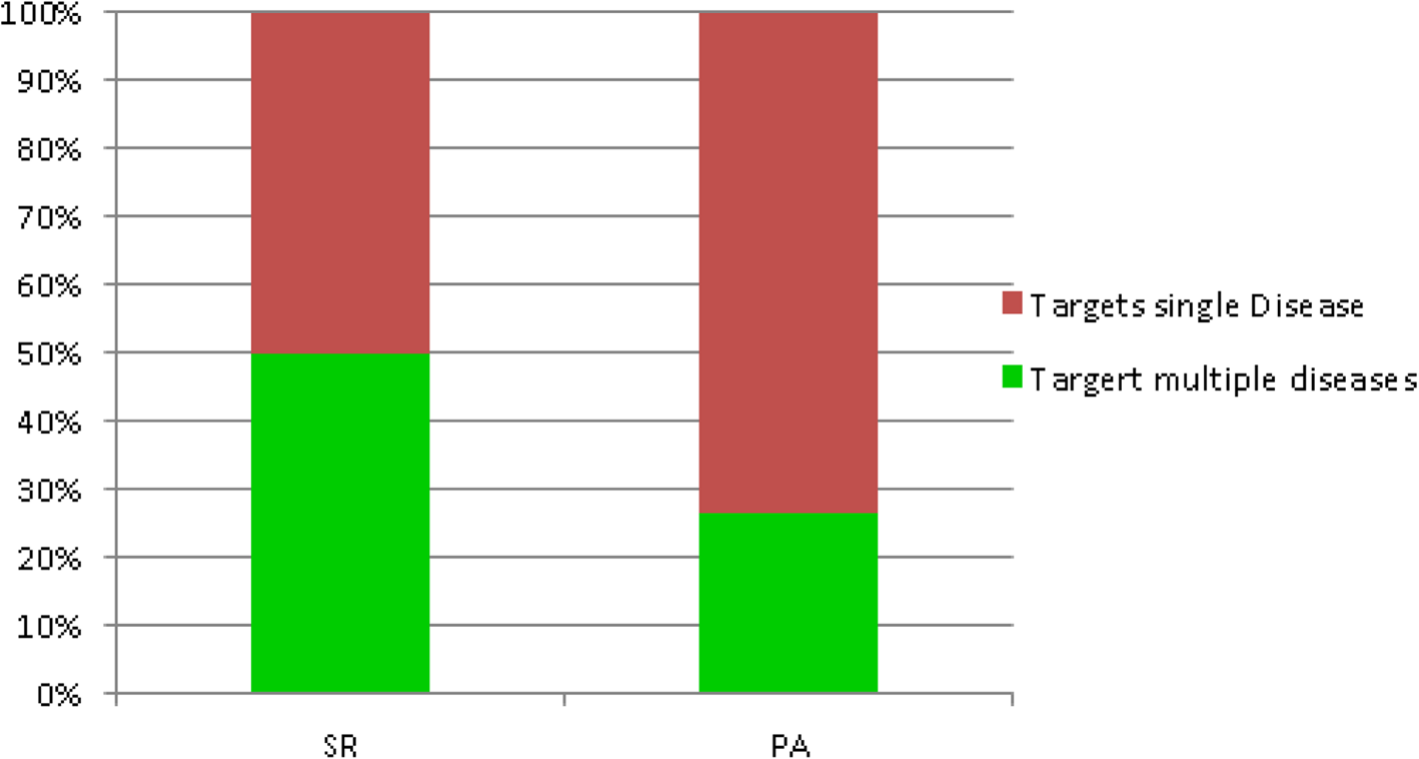
Comparison of targeted diseases between SR and PA

Systematic reviews generally discussed multiple diseases targeted with different, but often independent interventions. As demonstrated in Fig. 7, half of the reviews [2, 4, 11, 13, 24–26, 29, 41, 42, 44, 58, 59] discussed multiple diseases but were largely not specific to any one disease. Five [4, 11, 25, 41, 58] actually identified diseases they targeted by name and the remaining 8 were not specific to any particular disease. Of the above 8, three [4, 13, 24] involved audio or video conference engagements with patients, allowing them to discuss or target a broad range of unspecified conditions or diseases.

However, 8 reviews [9, 10, 12, 22, 27, 28, 45, 46] out of 26 targeted only one disease such as diabetes, COPD, or asthma, and 3 reviews [9, 22, 27] discussed interventions related to cardiovascular diseases. The remaining 5 reviews did not provide sufficient details to determine whether they targeted one or more diseases. Most of these simply monitored vital signs but were not clear about the targeted disease(s). Where multiple diseases were manifestly targeted in a particular systematic review, it was often not clear whether a combination of diseases was targeted by the same or different RPMTSs.

The picture was quite different with regard to primary articles (see PA in Fig. 7). Of the 30 articles reviewed, 22 articles [20, 30, 31, 33–36, 40, 47–54, 56, 57, 60–63] discussed interventions which targeted one, single disease. Targeted diseases ranged from PTSD, mental health, Parkinson disease and COPD to IBD and malaria. The remaining 8 articles [3, 15, 18, 32, 37–39, 55] tended to cover a combination of diseases but only three [32, 38, 55] were specific about the combination of diseases or parameters they sought to monitor or measure (HIV/AID and TB, and parameters related to CVD or COPD).

### Accessibility to the general public

**Fig. 8:**
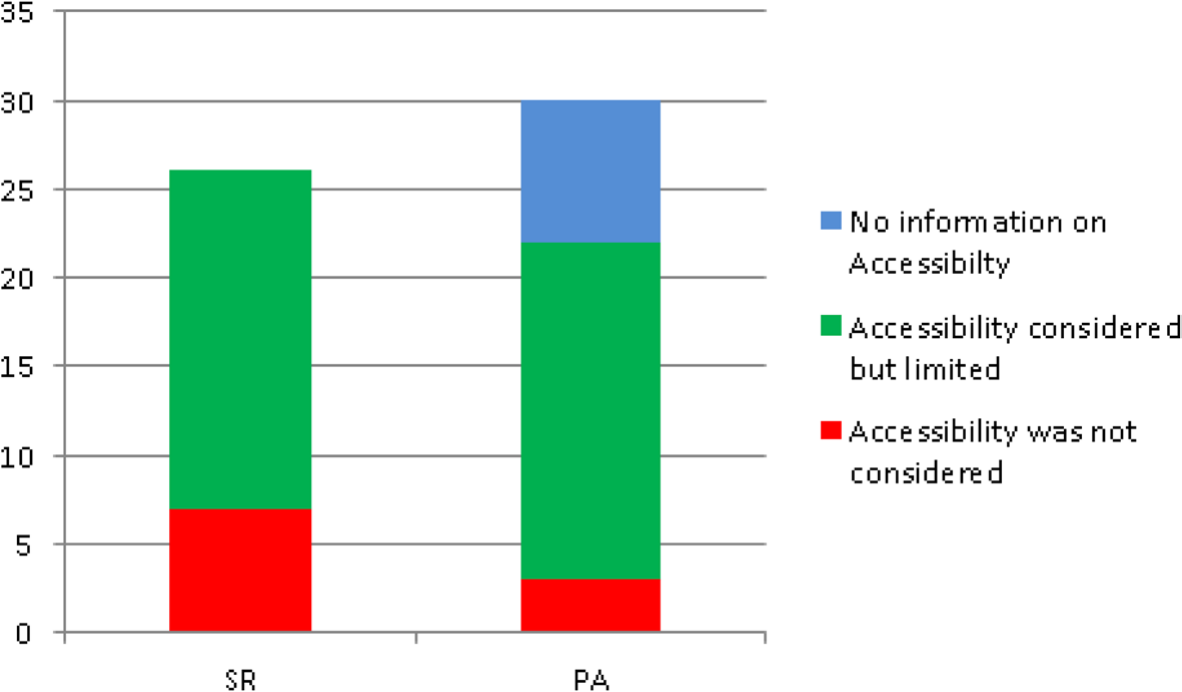
Levels of accessibility to the general public

While a number of systematic reviews did not provide enough details to determine the extent to which discussed interventions were accessible to the general patient and potential patient population, the majority [4, 10–13, 23–26, 28, 41, 43–46, 58, 59, 64, 65] provided information which indicated that accessibility was limited as depicted in Fig. 8. Among the many mentioned factors which negatively affect accessibility were usability, integration between patients’ home online systems and electronic health records, and giving personalized feedback [4, 58], centralized and decentralized data problem, which is a source of confusion and poses security and privacy challenges [26], performance in clinical settings which is still controversial [25] and insufficient healthcare infrastructure and funding [12, 24]. Other reviews however highlighted more systemic and historical challenges including those related to inequalities and the needs of the target user group which ought to be taken into consideration early in the design and development of mHealth tools; vulnerable, hard-to-reach or otherwise high-risk patient populations [11, 28, 43, 44, 59]; varying degrees of literacy, connectivity and accessibility and some patients who were concerned that their care would become dependent on technology, resulting in depersonalized care, reductions in face-to-face interaction and increased out of pocket costs [23, 64]; and characteristics of the care setting and circumstances surrounding individual patients such as rural vs urban, in or out-patient, care delivery and payment models, patient’s characteristics and care goals and preferences.

Studies of telehealth should consider combinations of apps of telehealth and outcomes that are important in these new models and that evaluate the specific contribution telehealth can make in these contexts [41] and review [17] pointed out that prior to deploying a newly developed intervention into healthcare settings, its practicality, clinical effectiveness and potential commercial benefits ought to be established and backed up by concrete evidence.

Primary articles also displayed similar results. Of the 30 articles, only three [34, 37, 39] provided information which indicated that accessibility of the intervention to the general public had been considered or was at least desired. Eight articles did not provide details related to accessibility. As depicted in Fig. 8, the majority of articles [3, 18, 30, 32, 33, 35, 36, 38, 40, 50–53, 55–57, 61–63] gave various reasons why accessibility of discussed interventions was limited including scalability [3, 30] health apps and smart phones’ credibility for continuous data flow, feasibility, portability and power consumption [33, 38, 53, 61], limited or lack of training [36], limited connectivity and Internet requirement of systems [35], and failure to take into account natural variations in patient physiology or behaviour [57]. Other mentioned factors were similar to those covered by systematic reviews.

### The main purpose of interventions

**Fig. 9:**
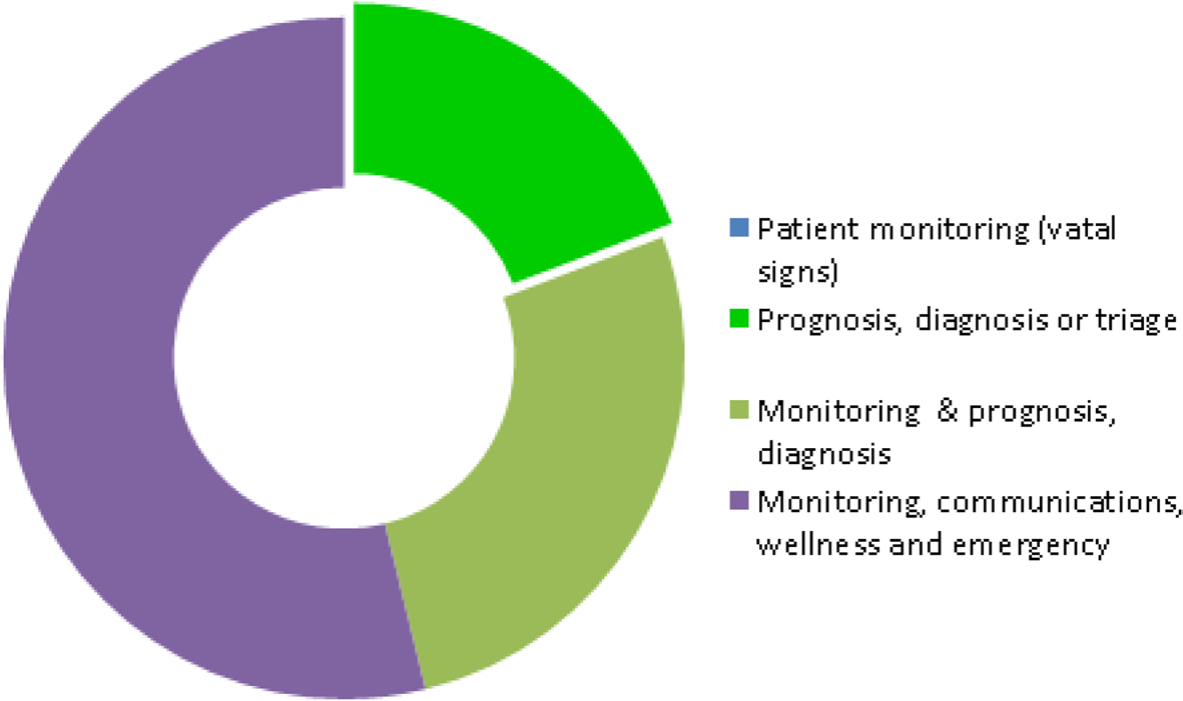
SR — healthcare organization’s main purpose

The large majority of systematic reviews discussed interventions which included patient monitoring for various purposes, ranging from reporting worsening symptoms of chronic diseases such as heart failure, COPD, asthma and infectious diseases to patient triage (see Fig. 9). In some cases [4, 22, 24, 26, 27, 29, 42], monitoring was combined either with prognosis or with diagnosis of various diseases. In relatively few cases [2, 12, 13, 23, 44], reviews discussed interventions which exclusively focused on prognosis, diagnosis or triage of patients without the requirement for continuous patient monitoring as part of the intervention. In the remaining 14 cases, reviews discussed interventions whose purpose was either a combination of communication, wellness and emergency alerts in addition to patient monitoring.

**Fig. 10:**
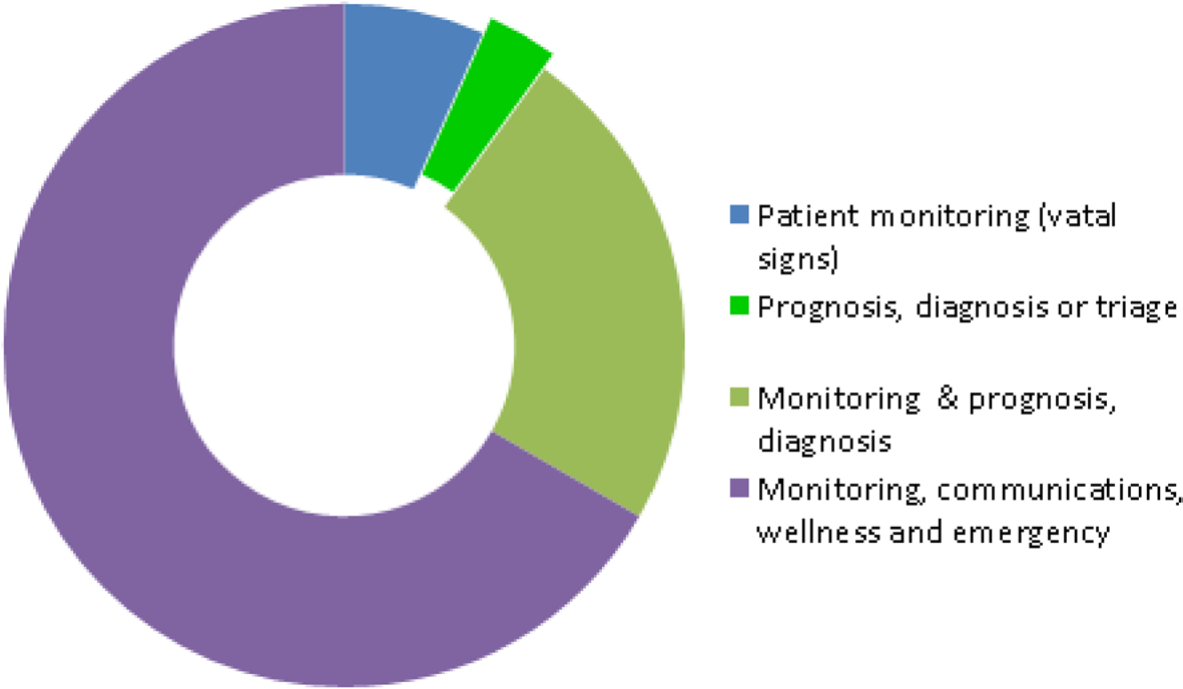
PA — healthcare organization’s main purpose

As far as primary articles were concerned, the vast majority of articles (20) discussed a combination of monitoring, communication, wellness and emergency alerts either for assessing the severity of symptoms of pre-existing health conditions or for managing patient’s adherence to treatment. However, as shown in Fig. 10, one article [3] discussed on-demand monitoring for triage purposes and only hinted at prognosis and diagnosis but did not clarify its level of integration with traditional healthcare systems. In the remaining cases [30, 31, 34, 35, 37, 39, 40, 60, 63], prognosis and/or diagnosis were mentioned along with continuous patient monitoring for vital signs. Overall, most articles were clear about their main purpose.

### Design and implementation approach

Of the 26 systematic reviews considered, only one review [10] discussed an implementation which placed patients at its centre, providing training, educational materials and daily phone calls to support patients. In seven reviews [2, 9, 26, 27, 58, 59, 65], the design was considered to be more technically centred, with patients and potential patients simply being expected to adopt the designed solution.

**Fig. 11:**
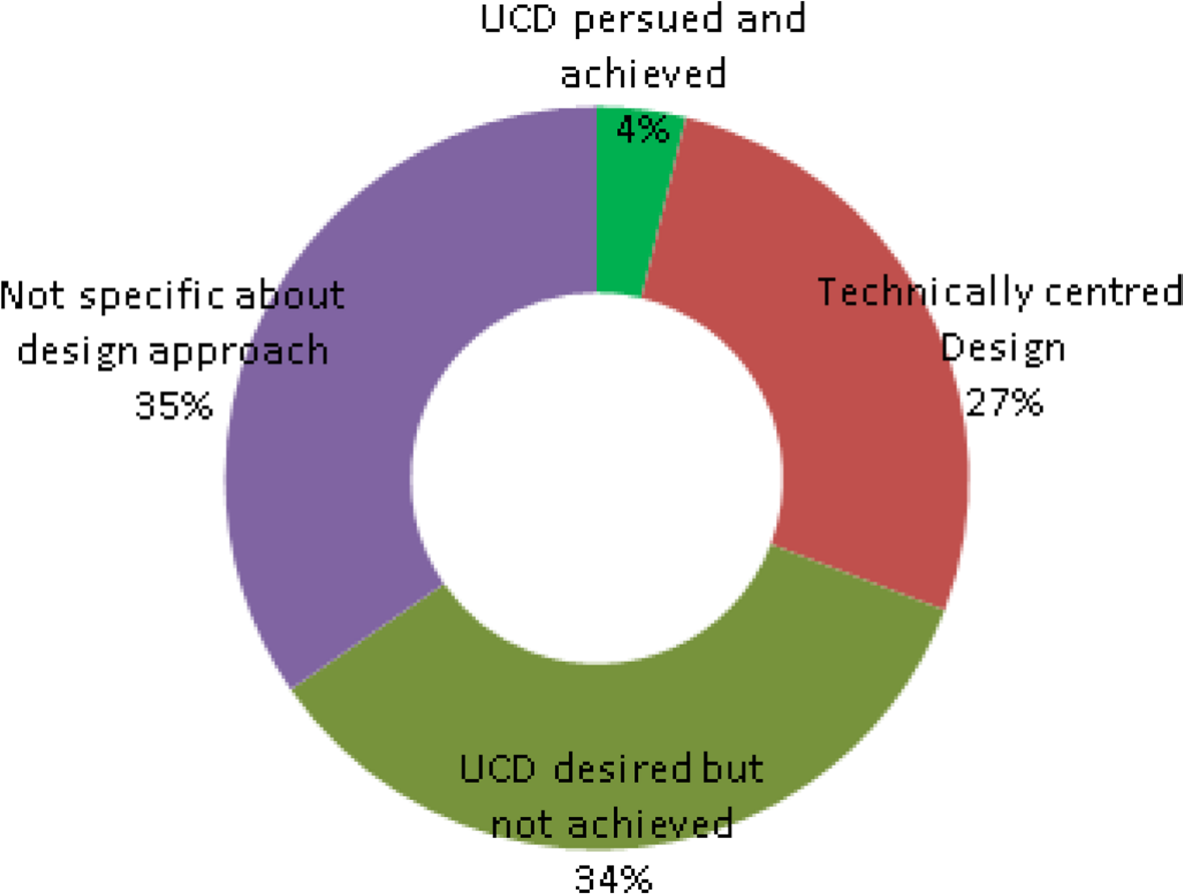
SR — design approach layout

Nine reviews [5, 22, 25, 28, 41, 43, 44, 46, 64] gave evidence of wishing to pursue a user-centred design but there was an indication that such design either was not achieved or was limited due to factors such as unavailability of mHealth apps on some operating systems [44], limited mobility and flexibility, in addition to the trustworthiness and quality of the content, and personalization possibilities through customization and adaptability [5] and usability drawbacks, as well as reports of the need for more comprehensive solutions, including the provision of real-time feedback and the integration of the EHR systems being used by the care providers [46]. The layout of design approach for systematic reviews is depicted in Fig. 11.

**Fig. 12:**
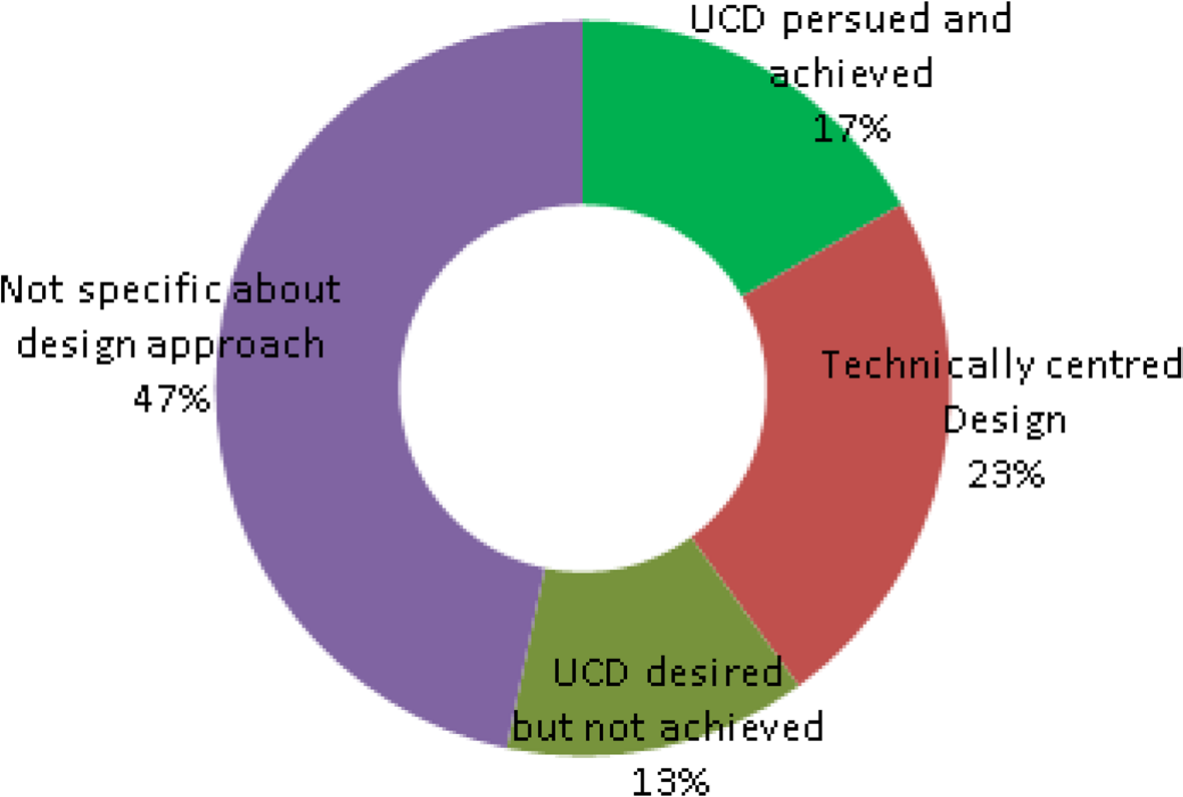
PA — design approach layout

With regard to primary articles, five [15, 20, 40, 53, 57] of all articles set out to pursue a user-centred design from the outset of intervention design by broadly consulting clinicians and patients. As can be seen in Fig. 12, seven considered articles [31, 33, 37, 52, 56, 61, 63] were deemed to have pursued a technical rather than a user-centred design approach and several of the considered articles discussed off-the-shelf solutions which required customization. However, even though most of the articles were silent about the design approach overall, the importance of a user-centred or patient-centric approach was broadly acknowledged to facilitate adoption by end-users, in the vast majority of reviewed primary articles.

## Analysis and discussion

A summary of findings along each of the six dimensions of analysis is provided in Table 3 along with observations and implication for the proposed framework and future research directions. From our scoping review of the landscape of RPMTS’s design, development and deployment, it appears that the majority of RPMTS interventions deployed in the pre-clinical setting for preventive purposes and known as ‘self-diagnosing apps or symptom checkers’ have not been found to be integrated into traditional clinical workflows. These interventions [22, 23, 28, 31, 36–39] are mainly deployed to monitor patients’ physical activities (PA) to address lifestyle-related diseases such as cardiovascular diseases (CVD), high blood pressure and diabetes in the context of the new ‘quantified self’ movement.

**Table 3:** Summary of evidence (findings and implications)

Dimension	Findings	Observations and implications for research
**Position**	The majority of RPMTSs are deployed in post-hospital settings to monitor chronic conditions, previously diagnosed within hospitals. Few RPMTSs are deployed in pre-clinical settings for preventive, prognostic or diagnostic purposes.	While the increasing prevalence of chronic diseases in ageing populations is the main driver for the rapidly increasing use of RPMTSs, deploying RPMTSs in remote, automated prognosis, preliminary diagnosis and prescheduling of visits to healthcare facilitates have significant potential for the prevention and early detection of above diseases and therefore ought to receive adequate research attention.
Levels of **integration**	RPMTSs deployed in post-hospital settings are generally integrated into existing clinical workflows. However, RPMTSs deployed in pre-hospital and primary care settings are often not integrated into existing clinical workflows (e.g. quantified-self apps).	RPMTSs can only help reduce the care burden on traditional healthcare systems when they are linked to them. There is therefore a need to consider integration into existing clinical workflows as a key requirement when designing RPMTSs for deployment in pre-clinical and primary care settings.
Functional **versatility**	While RPMTSs used in the management of chronic diseases are mostly targeted at a single disease and its related symptoms and vital signs, the few RPMTSs found in pre-clinical settings are generally versatile and tend to focus on a combination of potential diseases.	Addressing multiple diseases with a single RPMTS intervention might improve its likelihood for adoption and potential for scaling. There is a need for increased built-in, interpretive capacity to avoid expecting untrained users to make sense of resultant information on their own, without the necessary skills to do so (automatic interpretation of medical data is critical).
**Accessibility**	Accessibility is generally limited: Interventions in the preclinical and primary care settings are severely hampered by the lack of legal frameworks as well as issues related to information privacy and security and those in post-hospital settings for the monitoring of chronic conditions generally focus on a single disease, thereby limiting the number of potential adopters.	In post-hospital settings, the focus on a single disease means that only patients who suffer from the targeted disease can be addressed, thus limiting the scope for adoption and scaling. For RPMTSs contemplated for pre-clinical settings, there is a need to work with policy-makers to develop a legal framework and policies not only to address ethical and safety issues but also those related to information privacy and security.
Main intervention’s **purpose**	Healthcare organizations are mainly driven to utilize RPMTSs to manage the increasing care burden resulting from the rapid rise in chronic conditions. They are mainly used in an attempt to reduce the resulting skyrocketing care costs around the world. Improved care quality is also often targeted	The end-goal is not management but cost and workload reduction. Prevention could be less costly than treatment. By using RPMTSs to boost disease prevention and early detection, some diseases might be entirely avoided and the costs of managing chronic conditions might be significantly reduced.
Main **design approach**	The benefits of a user-centred or patient-centric design approach are widely acknowledged to promote adoption and scaling. However, in less than half of RPMTSs’ design cases, a user-centred or patient-centric approach is pursued and appropriate methods of involving users in RPMTS’s lifecycle phases are still in their infancy.	Involvement of users in the conceptualization, design and deployment of a new RPMTS is a key driver for its subsequent adoption, scaling and sustainability. Therefore, designers interested in the adoption and scaling of their RPMTSs ought to find a systematic way or method of allowing users to shape the design and deployment of their contemplated RPMTSs.

Reviewed literature, however, indicates that individual users/potential patients are often not able to make sense of the resultant health information to assess its applicability and impact on them, which could negatively affect their overall wellbeing and general health [66]. An integrated framework could therefore not only help facilitate the integration of the quantified self’s tools and symptom checkers into existing clinical workflows but also increase their functional utility to traditional healthcare systems to reduce the care burden thereto.

The literature on the design and deployment of RPMTS shows that these interventions do not consistently follow ‘a user-centred approach’ which is in line with the fragmented [15], direct to consumer models used to target those who are interested in their physical fitness and wellbeing [23]. With regard to above initiatives and their potential to improve health outcomes, Cornet and Holden [22] recommended that specialists (e.g. informaticists, computer scientists, etc.) should collaborate with clinical experts to identify and address problems amenable to passive sensing and indicated that only through these kinds of partnerships can novel technologies be designed and assessed for practical value, scalability and sustainability. Thus, an organizing framework to promote these collaborative engagements could contribute to the increased adoption and scaling of RPMTSs.

RPMTS interventions deployed in the pre-clinical setting also suffer from a lack of clear legal and policy frameworks to guide their design and deployment. For example, Hoffman et al. ([40], p. 2) point to perceived barriers which ‘can include concerns about privacy, legal ramifications, cost, workload, and need for increased information technology (IT) support’. The above reality could be the logical reason for Lobeloa et al. ([32], p. 10) to have concluded their systematic review by recommending that stakeholders should ‘work collaboratively to address privacy/security concerns and standardize frameworks to ensure reliability, validity and utility for PA promotion and CVD risk reduction applications in clinical and community settings, as well as population health management and public health advancement.’ Therefore, notwithstanding other possible limitations, legal and policy gaps along with the currently fragmented, direct to consumer deployment models may partially explain the absence of integration of these types of RPMTSs into traditional healthcare systems.

The proposed framework would aid in bridging above gaps and fragmentation especially in remote, automated prognosis, preliminary diagnosis and prescheduling of health-related appointments away from healthcare facilities. Indeed, as posited by Jacob, Sanchez-Vazquez, and Ivory, mHealth systems ought to continue to ‘help shift the focus of health care to a more patient-centric model that goes beyond treating diseases to a more predictive and preventative approach’ ([5], p. 2).

While the majority of RPMTS deployed in pre-clinical settings for preventive purposes were generally versatile and could gather symptoms about different diseases, it appears that users and their physicians are expected to discern the health implications for the generated health-related information which inadvertently increases the burden on patients/potential patients and their clinicians. Yet, as pointed out by Walker et al., technology should ‘be designed to have minimal user burden, be user-friendly, and have mechanisms installed to provide reassurance of safety’ ([14], p. 84). The advent of a well-integrated framework could encourage designers and developers of RPMTSs to avoid placing unnecessary burdens on users by paying careful attention to above critical drivers of adoption and scaling.

On the clinician’s side, Jacob, Sanchez-Vazquez and Ivory ([5], p. 16) concluded that ‘integrating mHealth in the clinical workflow is key to avoid that the tools become more of a hurdle to the staff’. Furthermore, speaking about difficulties encountered by clinicians in triaging patients for care, Kalid et al. ([3], p. 11) observed that ‘the overwhelming heterogeneous data cause difficulty in deciding which patient out of many should be provided with care first’ and concluded that ‘decision-based methods for prioritising patients in this environment are of urgent concern’ and Totten et al. [41] identified triage in urgent/primary care settings as a potential area of primary research to assist clinicians in their work. Therefore, designers of RPMTS ought to pursue completely automated, value adding processes and tasks which require minimal or no user and/or clinician’s involvement or intervention. The proposed framework could foster the above efforts.

However, for emergency, hospital and post-hospital settings, it was found that RPMTS generally focus on a single disease in the context of continuity of care (in home settings). In fact, Gray et al. found that some of the key weaknesses identified in Canada’s eHealth programmes to support people with complex care needs included the fact that ‘most technologies focus on single disease populations with few meeting the needs of “high users”, and there are few instances of links between healthcare and social-care organizations, making it difficult to wrap a full range of services and support around individuals who need them’ ([18], p. 31).

Targeting RPMTS initiatives to individuals who might need the same bundle of healthcare services, however, might require the design and deployment of RPMTS solutions for specific locations or areas in which given types of diseases are prevalent to meet the requirement of high number of potential users rather than focusing on specific, single diseases and struggling to achieve broad adoption. It is evident that the number of diseases targeted by any one, specific RPMTS intervention has a bearing on the general public’s accessibility to that intervention and ultimately affects its adoption and potential for scaling. As suggested by the reviewed literature, more than 70% of RPMTSs target a single disease and are deployed in post-hospital settings to address the growing list of complex, chronic diseases and disabilities.

The general public’s accessibility to these interventions in these contexts is thus inherently constrained and limited to those patients who suffer from the targeted disease and would naturally result in limited adoption and scalability. Therefore, notwithstanding all other relevant factors, levels of adoption and scalability in single disease cases ought to be assessed in the context of the small population of concerned caregivers and patients who suffer from the targeted disease. In contrast, a move to address a combination of diseases, prevalent in a given, specific catchment area could potentially increase the much needed adoption and scalability of RPMTSs, especially in developing countries.

As concluded by Totten et al., going forward, new research efforts should focus on emerging models of care, particularly value-based models where the use of telehealth may improve the ability to share risk and attain better quality and related outcomes. ‘These studies of telehealth should consider combinations of applications of telehealth and outcomes that are important in these new models and that evaluate the specific contribution telehealth can make in these contexts’ ([23], p. 52). Since heeding the above conclusion might lead to increased accessibility, adoption and scalability of RPMTSs in general, an integrated framework is needed to help design and develop, community-driven, localized multi-functional RPMTSs.

Reviewed literature has expressed support for patient-centric or user-centred RPMTS’ design, development and deployment approaches. Yet, more than half of the reviewed RPMTS interventions did not give evidence of having pursued a user-centred approach leading to a significant gap between the features and functionality that target users can easily embrace and features actually included in existing RPMTSs. To highlight the existing gap between users or patients’ real needs and existing RPMTSs, Rudin et al. ([56], p. 1032) observed that ‘of the more than 165,000 mHealth apps available, many have low usability, do not provide clinical utility, have minimal uptake, or are abandoned soon after first use. Few are designed to be integrated into clinical workflows, even those that are highly rated. Little is known about how to develop mHealth functionality that will not only provide clinical utility for chronic condition management but also will be adopted and used by patients and providers’. Above reality may partially explain the current low rate of adoption of RPMTSs, despite existing enthusiasm and hype around these interventions. It would thus be a good idea to further explore and possibly embrace the 3 key lessons set out by Smaradottir, Gerdes and Fensli ([20], p. 358) which are that ‘intended solutions for medical environments necessarily need to firstly involve all the user groups in the creation of the solution’.

Secondly, the respective analysis of how this solution could best fit in an existing clinical workflow or, if non-existent, embedding the solution in a new workflow built up in collaboration with the end-user groups.

**Fig. 13:**
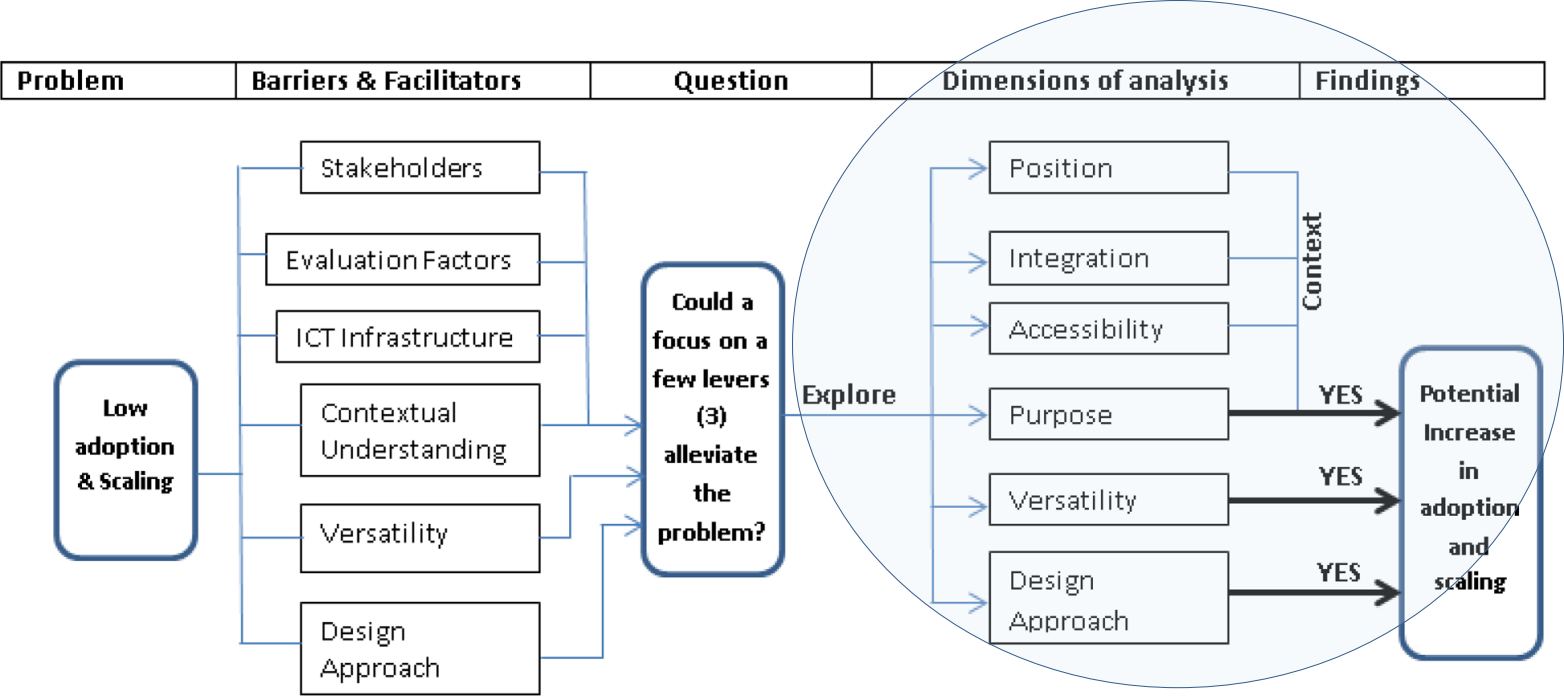
Summary concept map for analysis

Thirdly, the reality that chronic patients do not have the same levels of physical energy as healthy people underlines the importance of designing easy-to-use solutions that minimize physical effort and mental workload. Therefore, from the above discussion, the application and use of a user-centred, context-dependent, customizable framework focusing on a combination of diseases prevalent in a given catchment area could enhance improved design and deployment of RPMTSs and increase their adoption and scaling, especially in primary care contexts. Figure 13 depicts the conceptual analysis leading to these findings.

## Limitation of the review

This review has significant limitations. First of all, only one researcher did the refinement of the research question, data extraction and the synthesis of evidence. Secondly, the researcher in question did not review the academic quality and rigour of the included and reviewed articles. It should also be noted that reviewed literature is not necessarily statistically representative of the healthcare landscape in all countries.

Interestingly, Kristoffersson and Lindén [27] who also very recently reviewed literature on the use of wearable body sensors for health monitoring highlighted a number of signification shortcomings in published articles between 2010 and 2019, including small sample sizes (only 20% of the studies involve more than 100 participants), poor presentation (not providing enough or sufficient information on how the experiments were conducted) and using non-representative participant demographics or not providing information on representative participant demographics (age, gender, patient/healthy, etc.).

In this particular scoping review, there has also been a deliberate exclusion of relevant articles published before 2012 for systematic reviews and those published before 2014 for primary articles. Furthermore, the explosive increase in the amount of literature covering the topic of COVID-19-related remote care solutions since May 2020 to date may have implications for this study. Even though growth in this area largely occurred in developed countries with existing technical and financial infrastructure to support remote care in general and in particular video and voice call consultations, there can be no doubt that there has been a general shift in the disposition to adopt and scale RPMTSs overall. However, it is not clear whether this momentum will be sustainable as the COVID-19 pandemic begins to subside. Considered articles also varied greatly in their nature and extent to which they provided insights into variables of interests and resultant implications for answering the research question of interest. Therefore, subjective extraction and evidence synthesis methods employed by the researcher may have impacted his perception and understanding.

All of these factors might indicate some degree of bias and may have significant implications for the validity of drawn conclusions and recommendations. Added to this are the limitations normally associated with scoping reviews. Some of these limitations are summarized in Table 4.

**Table 4:** General limitations of the scoping review

	Potential bias/issues
Review by one researcher	Reduced transparency and reproducibility
Only one reviewer extracting data	Increased risk of errors and missing key, relevant points
Considering only recent articles	Key articles and results could be excluded
Excluding non-English publications	Important studies/reviews in other languages may have been missed
Limited access to relevant databases	Key articles may not have been considered due to inaccessibility
Flexible review/study design	Reduced accuracy, validity and possible bias

## Conclusions and future work

The completed scoping review has suggested that identified levers may indeed play an important role in improving the adoption and scalability of RPMTSs. No single article discredited any of the identified levers (contextual understanding, combination of diseases in a given catchment area and a user-centred design approach), while the vast majority of reviewed publications pointed to the importance and utility of the proposed framework to entrench identified levers in the conception, design, development and deployment of RPTMSs. Therefore, based on reviewed, recently published material, it seems likely that the application and use of a user-centred or patient-centric, context-dependent, customizable framework may assist in increasing the adoption and scalability of RPMTSs, if such framework addressed a combination of diseases, prevalent in a given, specific community or catchment area.

Although reviewed publications did not directly address the process through which the proposed framework would improve RPMTS’s adoption and scalability, this review has shown that such a framework would be able to assist RPMTS’s designers and developers to address issues most likely to influence adoption and scalability during and prior to planning and designing such RPMTSs, by carefully studying the contexts within which prospective RPMTSs will be subsequently deployed and by engaging target users throughout an RPMTS’ intervention lifecycle. The extent of the increase in adoption and scalability was not addressed but would obviously depend on how well target users and their specific contexts are understood by RPMTS’s designers and developers.

Based on the above conclusions and previously stated limitations of this review, it is recommended that future research efforts be directed towards the design of a research study (or studies) to develop the proposed framework and determine how such a framework, once developed, can be tested and validated in the field to achieve desired outcomes.

## Appendix 1

Table 5

**Table 5:** How barriers and facilitators affect the adoption and scaling of RPMTSs

Nr	Main categories	Sub-categories (potential barriers and facilitators)	How sub-categories affect adoption and scaling of RPMTS interventions	Secondary source used
1	***Stakeholders’ interests***			
	**Conflicting and dynamic interests of and relationships between stakeholders are sure to have a significant impact on the adoption and scaling of RPMTSs. The extent to which RPMTS designers and developers are able to*****manage*****these conflicts and power relations may have a bearing on adoption and scaling thereof.**	Healthcare organizations	Pursued visons, missions, strategies, funding structures and profit motives determine organizational structures and priorities. This may in turn lead to resistance or commitment to a particular RPMTS intervention.	[5, 22]
		Clinicians	How a new RPMTS intervention will affect existing workflows, work dynamics and job security will lead to support or resistance by clinicians and other healthcare practitioners.	[5]
		Patients or potential patients	Incentives such as being able to save on money and/or time while enjoying improved quality of care and better access to healthcare services may promote adoption and/or scaling.	[4, 5]
		Health technology companies	The visons, missions, strategies and profit motives will affect how RPMTS interventions are designed and deployed (intellectual property, policies and regulations) thereby positively or negatively affecting adoption and scaling.	[22]
		Governments	Government’s political priorities, policies and regulations may significantly promote or stifle an RPMTS’s development and potential for adoption and scaling.	[5, 22]
		Others (investors, NGOs, etc...)	Interests of other institutions such as NGOs, professional associations and lobby groups expressed in their missions and goals may promote or hinder the development, adoption and scaling of certain RPMTS interventions.	[22]
2	***Contextual understanding***			
	**The deeper the understanding that RPMTS designers and developers have of the*****context*****within which their planned intervention will be deployed, the more likely the intervention is to be suitable for adoption and scaling.**	Community’s socio-economic factors	A community’s economic status and general social realities (income levels, social cohesion, financial resources...) can impact the potential for adoption and scaling of an RPMTS.	[4, 5, 22]
		Socio-cultural, values and beliefs	Cultural beliefs and values espoused by a given target community may lead to resistance or acceptance of an RPMTS’s intervention.	[4, 5, 22]
		Political priorities	Prevailing political views and priorities may enhance or hinder the development, adoption and scaling of RPMTS interventions.	[22]
		Health standards, policies and guidelines	Existing health policies, standards and guidelines may allow and encourage or obstruct the development, adoption and scaling of RPMTS interventions.	[4, 5, 22]
		General attitude towards technology	A community’s general interest in and experience in technology use (such as the use of smart phones and related apps) may be indicative of its propensity to adopt or not adopt RPMTS interventions.	[4, 5]
		Levels of education and technology skills	General levels of education has a bearing on the ability of a community to grasp the benefits of RPMTS’s use and to therefore take advantage of available learning and training opportunities around RPMTS interventions.	[4, 5, 22]
3	***Existing local ICT infrastructure***			
	**Prior to the planning and designing of an RPMTS intervention, adequate ICT infrastructure (appropriate network coverage, device penetration, data costs and reliability thereof) must exist to support its deployment and subsequent scaling.**	ICTs’ accessibility, availability and sustainability	A community’s accessibility to an ICT infrastructure with long-term financial sustainability (costs of data, apps and devices) may have a significant impact on RPMTS’s adoption and scaling.	[4, 5, 22]
		Connectivity and reliability	The reliability and stability of established ICT connections for the purpose of healthcare services increase the community’s trust and confidence in RPMTS’s interventions and their potential to effectively complement or replace face to face service.	[4, 5]
		Potential for stakeholder collaboration	The ability of stakeholders to collaborate within and across industries to achieve health goals (e.g. ICT providers’ willingness to reduce data costs used for health purposes) may significantly improve the adoption and scaling of RPMTS interventions	[5, 22]
		Adequate technical support	The availability of adequate technical support increases the sustainability, continued adoption and scaling of RPMTS interventions. New users may adopt a new RPMTS intervention because of the availability of reliable, adequate support.	[4, 5]
		Network capacity and device penetration	The prevalence of mobile devices (smart phones) and network capacity in the target area may limit the potential for scaling and further adoption of a given RPMTS intervention.	[5, 22]
4	***Design approach***			
	**When the design of an RPMTS intervention is*****centred on*****its targeted users, their needs and lifestyles, they will be more inclined to adopt it. Furthermore, if they are engaged in shaping it*****from its inception*****, they may feel a sense of ownership, which may positively influence their attitude toward its adoption and scaling.**	Interoperability and compatibility	The extent to which new RPMTS interventions seamlessly fit into, interface and work with and within existing healthcare systems has a significant impact their adoption and scaling.	[4, 5, 22]
		Patient-centred design	The extent to which RPMTS interventions are designed to meet the needs of and provide tangible benefits to patients and potential patients significantly increases the chances of adoption and scaling of RPMTS’s interventions.	[4, 5]
		Functionality and adaptability	The inclusion of features and functionalities which are in light with the needs of intended users as well as the potential for customizing above features to a broad range of user groups would foster increased adoption and scaling of RPMTS interventions.	[4, 5, 22]
		Integration in clinical workflows	Integration of RPMTS interventions into clinical workflows and EPR, EMR and EHR improves access to and quality of healthcare services and may lead to their increased adoption and scaling.	[4, 5]
		Collaboration across the healthcare domain	Coordination of health services and collaboration between healthcare professionals helps to align often conflicting interests and may promote improved adoption and scaling of RPMTS interventions especially among clinicians.	[5, 22]
		User engagement	Involvement of users in development and planning of RPMTS interventions allows planners to become better acquainted with their requirements and to become aware of their potential resistance to adoptions and scaling of RPMTS interventions.	[4, 5]
		Simulation and validation (triability)	Opportunities to learn about and try RPMTS interventions without strings attached may increase the trustworthiness of specific RPMTS interventions and hence increase adoption and scaling.	[5]
		Fit between technology, users and organization	The extent to which RPMTS interventions are aligned with healthcare organizations’ goals and missions and helps users achieve their objectives (e.g. cost-effective, quality care) may determine their adoption and scaling.	[5]
		Data privacy and security	Privacy and security issues and concerns related to an RPMTS intervention may limit or even prevent its adoption and scaling altogether as potential users are not prepared to compromise their privacy.	[4, 5, 22]
5	***Triggers for use and adoption***			
	**Interventions ought to provide*****use triggering opportunities*****to potential users to try or start using a particular RPMTS intervention. The greater the number of people in the targeted community able to come across and access these opportunities, the more likely an RPMTS intervention is to be adopted and scaled.**	Number of targeted diseases	The diversity of health conditions addressed by an RPMTS intervention broadens opportunities for its uses (or usefulness). Furthermore, it may be the case that the greater the number of its users, the lower its costs per a user (economies of scale).	[4]
		Awareness and promotion	The extent to which new RPMTS interventions are promoted can shape attitudes and perceptions of potential users and trigger subsequent adoption and scaling of these systems and tools.	[5, 22]
		Trustworthiness and quality	The quality and trust that potential users perceive and experience from an RPMTS intervention may be a key trigger for its subsequent use, adoption and scaling.	[4, 5]
		Ease of use and automation	The ease of use and level of automation of RPMTS interventions can encourage users to start using them and eventually lead to their adoption and scaling.	[4, 5]
		Mobility and flexibility	Mobility and flexibility offers convenience to potential users and may help trigger the adoption of RPMTS intervention and lead to their subsequent scaling.	[4, 5]
		Training (clinician and users)	Opportunities for training on new RPMTS interventions often triggers adoption and may lead to subsequent scaling of these interventions.	[4, 5]
		Promotion of self-management	Promotion of self-management empowers clinicians and patients and increases their sense of ownership of an intervention, leading to adoption and subsequent scaling.	[5, 22]
		Accessibility to the general public	All things being equal, a more easily accessible RPMTS intervention is more likely to be used than one the public struggles to access or one which only a small number of people can access.	[4, 5]
		Perception and short feedback times	If users perceive RPMTS interventions as proving them with quick feedback than traditional channels, they are more likely to try them and adopt their use. Potential for scaling is also increased.	[4, 5, 22]
		Ability to complement or replace visits to clinics	Users who feel that RPMTS interventions complement or can replace face-to face interventions will be motivated to use them when accessing healthcare services.	[4, 5]
6	***Post-deployment assessment factors***			
	**Interventions are expected to be able to convincingly demonstrate (*****a-priori*****) evidence of how they will be able to meet certain*****key performance indicators after*****their implementation in order to attract adequate support and funding which may in turn have a bearing on their subsequent adoption and scaling.**	Reduced healthcare costs	If stakeholders and users believe that the use of RPMTS leads to reduced healthcare costs, they are more likely to promoted its adoption and scaling.	[4, 5, 22]
		Return on investment (funding)	Funders expect some form of return on their funds and the extent to which an RPMTS intervention can demonstrates its sustainable benefits in this regard, the more likely that the necessary funds will be made available to design them for adoption and scaling.	[5, 22]
		Better quality of care	Planned RPMTS Interventions able to demonstrate evidence of improved quality of care after their deployment are more likely to improve their chances of receiving adequate funding and subsequent adoption and scaling.	[5, 22]
		Reduced rates of hospitalization	RPMTS Interventions capable of demonstrating reduced rates of hospitalization are not only more likely to attract adequate funding but also likely to be adopted and scaled	[4]
		Community wellbeing	RPMTS interventions which emphasize the relationship between healthcare providers and the community they serve to promote overall community’s wellbeing are likely to be adopted and scaled.	[22]
		Reduced waiting times and overcrowding	Patients and potential patients are likely to adopt RPMTS interventions which reduce their waiting time and clinicians may promote those the reduce overcrowding at their health facility.	[4, 5, 22]
		Improved access to healthcare services	RPMTS interventions demonstrating evidence of improved access to healthcare services (without increasing the care burden on traditional healthcare systems) after their implementation are more likely to be funded, adopted and scaled.	[4, 5]

## Appendix 2

Table 6

**Table 6:** Summary of the characteristics of the included studies

Key variables	Subthemes	References
Position	***Systematic reviews***: communication between clinicians and patients prior to visits, telemedicine, detecting or predicting health deterioration, e-consultation; collection and tracking health-related data, prehospital assistance, symptom checkers, and community workers with health apps	[4, 12, 27–33, 66]
	***Primary articles***: remote monitoring in primary care, classification and prediction, behavioural health services in primary care, Primary Care-Mental Health Integration, real-time visualization, quantified-self, technology-based objective measures, diagnosis of infectious diseases, digital cryptocurrency and blockchain, referrals through a smartphone app, preventive care, health awareness, self-monitoring and community-based disease surveillance	[3, 34–44]
Integration	***Systematic reviews***: complimentary monitoring tool, users behaviours and motivations, clinical communication, HIS clients, medical training, prompt medical care under emergencies, telehealth, telemedicine and video conferencing	[4, 9, 12, 23, 27, 29, 45, 46, 48]
	***Primary articles***: integration of behavioural health, collaborative team building, remote monitoring and communication, reporting to the dispensary for laboratory diagnosis, Fracture Liaison Service Model of Care, remotely sent data to the hospital, data is embedded on web-based TDS platform, RPM involves patients/caregivers in their care, tablets transmit data to a remote secure cloud, and alert activated when symptoms are reported	[20, 35, 39, 40, 50–55]
Versatility	***Systematic reviews***: CVD include hypertension (HTN), coronary artery disease, and congestive heart failure, fall detection, chronic lung diseases (CLDs) include asthma and chronic obstructive pulmonary disease (COPD), brain and neurological disorders, mental health, diabetes, cancer, burn injuries, cognitive rehabilitation, HIV, multiple sclerosis, PD, physical activity, psychotherapy, cardiac, epilepsy, dementia, or paralysis disease, perinatal depression, high-risk pregnancy, foetal and pediatric cardiology, pre-eclampsia, pregnancy termination, and foetal alcohol spectrum disorder	[4, 10, 23, 29, 61]
	***Primary articles***: triage (vital signs and features), multiple sclerosis, low back pain, and osteoporosis, COPD, seasonal affective disorder (SAD), and bipolar affective disorder (BAD), Heart disease and HIV/AIDS and TB	[3, 18, 36, 41–43, 58, 64]
Accessibility	***Systematic reviews***: telehealth, telemedicine, video conferencing and home online Health Consultation	[4, 12, 23, 27]
	***Primary articles***: positive evaluation of the app, incentives for use (additional functionality) and unlimited access to the system	[38, 41, 43]
Main purpose	***Systematic reviews***: early detection and the management of chronic conditions, accessed from any web enabled smartphone, e-consultation and AI diagnosis	[2, 11, 12, 48, 66]
	***Primary articles***: improving the effectiveness and efficiency of prevention and prediction	[3]
Design approach	***Systematic reviews***: consultation sessions, training, and follow up phone calls (during deployment)	[9]
	***Primary articles***: doctors and nurses participated in each stage of its design and prototyping, Patient representative on the research team, patient acceptability before and after design, user centred design and feedback from the clinical collaborators and the patients in design	[20, 44, 56, 60, 64]
